# Selective decontamination regimens in French ICUs: author’s response

**DOI:** 10.1186/s13613-025-01571-8

**Published:** 2025-09-25

**Authors:** Nicolas Massart, Marc Leone, Alain Lepape, the REAREZO Study Group

**Affiliations:** 1 Service de Réanimation Polyvalente, Centre Hospitalier de Saint Brieuc, 10, rue Marcel Proust, Saint-Brieuc, 22000 France; 2 Service d’Anesthésie et de Réanimation, Aix Marseille Université, Assistance Publique-Hôpitaux de Marseille, Hôpital Nord, Marseille, France; 3 MEPHI, IHU Méditerranée Infection, Aix-Marseille Université, Assistance Publique-Hôpitaux de Marseille, Marseille, France; 4 REA-REZO Infections et Antibiorésistance en Réanimation, Hôpital Henry Gabrielle, Saint-Genis-Laval, France; 5 Département d’Anesthésie Médecine Intensive Réanimation, Centre Hospitalier Lyon Sud, Hospices Civils de Lyon, 165 Chemin du Grand Revoyet, Pierre-Bénite, 69310 France; 6 Centre International de Recherche en Infectiologie, PHE3ID, Institut National de la Santé et de la Recherche Médicale U1111, CNRS Unité Mixte de Recherche 5308, École Nationale Supérieure de Lyon, Université Claude Bernard Lyon 1, Villeurbanne, France

Dear Editor,

We thank Bernard Allaouchiche for his relevant comment on our recent study entitled “Selective Decontamination regimens in French ICUs” [1, 2]. He appropriately questions the diagnosis criteria of ventilator associated pneumonia (VAP) in patients receiving selective digestive decontamination (SDD), reporting an aminoglycoside leakage in the patient’s blood. This issue has been reported in previous trials. For instance, Camus et al. reported that among 259 patients receiving a SDD regimen, it was discontinued in 21 due to serum concentration exceeding 2 mg/L [3]. Similarly, Oudemans-Van Straten et al. reported that most acute critically ill patients treated with SDD had traces of tobramycin in the blood, notably those with shock, inflammation and acute kidney injury although nephrotoxicity could not be demonstrated [4].

Regarding the impact of the systemic passage of aminoglycoside on VAP diagnosis, however, we respectfully do not share the same point of view. First, our study as the most recent randomized controlled trials on SDD focused on ICU-acquired infections, notably bloodstream infections which the diagnosis is not subjective. Second, independently of definitions, it seems to have an association between a decrease in bacterial resistance and the use of SDD, which is probably critical for our patients. Finally, usual microbiological criteria were retained based on historical cohort using post-mortem exam as gold standard [5]. Noteworthy, in this historical study, the authors reported that “although 15 patients (53%) were not on antibiotics or were off antibiotics for more than 48 h before testing, no relationship could be established between the patients’ antibiotic status and the result of any diagnostic test”.

To our opinion, until further research is conducted, antibiotic exposure should not modify usual microbiological criteria for VAP diagnosis. Whatever the debate around definitions, SDD was associated with less bloodstream infections, less emergence of resistant pathogens, and less exposure to mechanical ventilation, which is critical in terms of patient-reported outcome measures.

## Data Availability

Not concerned.
